# Autoantibodies to neurotransmitter receptors and ion channels: from neuromuscular to neuropsychiatric disorders

**DOI:** 10.3389/fgene.2013.00181

**Published:** 2013-09-20

**Authors:** Pilar Martinez-Martinez, Peter C. Molenaar, Mario Losen, Jo Stevens, Marc H. De Baets, Andrei Szoke, Jerome Honnorat, Ryad Tamouza, Marion Leboyer, Jim Van Os, Bart P. F. Rutten

**Affiliations:** ^1^Department of Psychiatry and Psychology, School for Mental Health and Neuroscience, Maastricht UniversityMaastricht, Netherlands; ^2^Pôle de psychiatrie, Hôpital Henri Mondor – Albert Chenevier – Assistance publique – Hôpitaux de Paris, Université Paris-Est CréteilCréteil, France; ^3^INSERM U955, Equipe 15 Psychiatrie GénétiqueCréteil, France; ^4^Fondation FondamentalCréteil, France; ^5^Centre de Référence Maladies Rares ‘Syndromes Neurologiques Paranéoplastiques’, Hospices Civils de Lyon, Neurological HospitalBron, France; ^6^Université Claude Bernard Lyon 1Lyon, France; ^7^Laboratoire Jean Dausset and INSERM, UMRS 940, Hôpital Saint LouisParis, France

**Keywords:** NMDA receptor, AMPA receptor, GABA receptor, glycine receptor, acetylcholine receptor, Caspr2, Lgi1, potassium channel

## Abstract

Changes of voltage-gated ion channels and ligand-gated receptor channels caused by mutation or autoimmune attack are the cause of so-called channelopathies in the central and peripheral nervous system. We present the pathophysiology of channelopathies of the neuromuscular junction in terms of loss-of-function and gain-of-function principles. Autoantibodies generally have reduced access to the central nervous system, but in some cases this is enough to cause disease. A review is provided of recent findings implicating autoantibodies against ligand-activated receptor channels and potassium channels in psychiatric and neurological disorders, including schizophrenia and limbic encephalitis. The emergence of channelopathy-related neuropsychiatric disorders has implications for research and practice.

Autoimmune diseases are estimated to affect between 7 and 9% of the general population (Cooper et al., 2009). They can show a wide expression of phenotypes ranging from endocrine, musculoskeletal to neurological, and neuropsychiatric syndromes. A number of central nervous system (CNS) disorders, such as limbic encephalitis, have been demonstrated to associate with specific serum autoantibodies. Antibodies that are directed against voltage-activated ion channels and ligand-activated ion channel neurotransmitter receptors can be expected to have an important impact on functioning of neurons in a neural network, possibly leading to serious mental disturbances. In this connection it is relevant to bear in mind that neurotransmitter receptor blockers such as phencyclidine (angel dust), quinuclidyl benzilate, and lysergic acid diethylamide (LSD), affecting NMDA, muscarinic acetylcholine (ACh), and 5HT receptors, respectively, are all potent hallucinogens. As far as the NMDA receptor (NMDAR) is concerned the blockers phencyclidine, ketamine and the drug MK801 have been shown to cause cognitive defects in various rodent studies (Jones et al., 2011; Wiescholleck and Manahan-Vaughan, 2013). Blockers of muscarinic receptors, e.g., scopolamine and pirenzepine, affect working memory of rats in maze tasks (Graef et al., 2011). Blockers of nicotinic receptors, mecamylamine and various synthetic drugs that are more or less specific blockers of α7 and α4β2 receptors, impair the performance of rats in a radial arm maze (Graef et al., 2011).

Receptors play an important role in functioning of the nervous system. Their essential functions are also illustrated by the high conservation of amino acid sequence, structure, and function of receptor gene families. Furthermore, several defects in ion channels cause impairment of neural function in both the peripheral and the CNS. Before we focus on recent developments in autoimmune channelopathies as a cause of mental illness in subgroups of patients, we start with a brief summary of neuromuscular channelopathies. This field of research has been exemplary for the study of individual ion channels or receptor channels with patch clamp electrophysiological methods and this information is likely to translate into insights about CNS channelopathies. In the neuromuscular system this approach has allowed for the identification of the pathophysiological mechanisms of many genetic or autoimmune defects of ion channels (Lehmann-Horn and Jurkat-Rott, 1999; Lorenzon and Beam, 2000; Striessnig et al., 2004; Cannon, 2010). In this review we discuss the evidence that autoantibodies in neuropsychiatric disorders cause predominantly a loss rather than a changed pattern of channel activity, possibly associated with neuroinflammation and neurodegenerative changes.

## LOSS-OF-FUNCTION AND GAIN-OF-FUNCTION CHANGES IN NEUROMUSCULAR CHANNELOPATHIES

Mutations can cause loss-of-function (L-O-F) either because the corresponding proteins are so much changed that they cannot be properly processed and incorporated into the membrane, or that they are dysfunctional in the case that they are incorporated. Such diseases are usually autosomal recessive because the other wild-type chromosome provides unaffected copies of the channel protein that may be enough to take over all necessary activity from the dysfunctional channels so that the overt disease phenotype is not expressed. This is the case with the chloride channel (Pusch, 2002; Ryan and Ptacek, 2010), potassium channels (Shillito et al., 1995; Irani et al., 2010), and ACh receptor (AChR) channels in muscle (Engel et al., 1999; Faber et al., 2009).

Voltage-activated channels open and close, and are inactivated and re-activated as a function of membrane voltage and time. Mutations occurring in the voltage-sensing part of the channel subunit can thus severely disrupt their finely tuned behavior (Cannon, 2010; Jurkat-Rott et al., 2010a). Autosomal dominant mutations, for example in genes encoding sodium channels, may induce the channel to inactivate too slowly after opening, leading to persistent depolarization of the membrane (Jurkat-Rott and Lehmann-Horn, 2004; Jurkat-Rott et al., 2010a,b). Such mutant channels with abnormal functional behavior result from gain-of-function mutations although the word “gain,” perhaps, might wrongly suggest that a mutation has caused the function to “improve.” On the contrary, action potential formation may be completely disrupted regardless of the fact that properly functioning ion channels are still expressed by the wild-type gene. This is the case with sodium channel diseases in muscle (Jurkat-Rott et al., 2010a) and in the slow channel syndrome with mutations of the AChR where the channels stay open too long (Oosterhuis et al., 1987; Engel et al., 1996).

**Table 1** gives a list of the diseases that are caused by channel dysfunction in the neuromuscular junction. A number of general features emerge from research into these diseases:

**Table 1:** Neuromuscular channelopathies.

Membrane protein	Disease	Symptoms	Subunit/associated protein	Functional change^#^	Gene or antibody defect	Reference^*^
**Skeletal muscle**
Sodium channel	Hyperkalemic periodic paralysis	Myotonia and or weakness	Na_v_1.4	G-O-F	SCN4A	Jurkat-Rott et al. (2010a), Ryan and Ptacek (2010)
	Paramyotonia congenita	Stiffness and weakness	Na_v_1.4	G-O-F	SCN4A	Jurkat-Rott et al. (2010a), Ryan and Ptacek (2010)
	Hypokalemic periodic paralysis	Weakness	Na_v_1.4	G-O-F	SCN4A	Fontaine et al. (2007), Cannon (2010), Jurkat-Rott et al. (2010a)
Potassium channel	Thyrotoxic hypokalemic periodic paralysis	Weakness	Kir2.6	G-O-F	KCNJ18	Ryan et al. (2010)
Calcium channel	Hypokalemic periodic paralysis	Weakness	Ca_v_1.1	G-O-F	CACNA1S	Lorenzon and Beam (2000), Striessnig et al. (2004), Fontaine et al. (2007)
Chloride channel	Myotonia congenita Thomsen^1^	Myotonia	ClC-1	L-O-F	CLCN1	Kullmann and Waxman (2010), Ryan and Ptacek (2010)
	Myotonia congenita Becker^2^	Myotonia	ClC-1	L-O-F	CLCN1	Kullmann and Waxman (2010), Ryan and Ptacek (2010)
Nicotinic receptor	Congenital myasthenic syndrome	Weakness	n-AChR, α	L-O-F	CHRNA1	Engel et al. (1999)
			β		CHRNB1	
			δ		CHRND	
			ε		CHRNE	
			rapsyn		RAPSN	
	Slow channel syndrome	Weakness	n-AChR, ε	G-O-F	CHRNE	Engel et al. (1999)
	Myasthenia gravis	Weakness	n-AChR, α	L-O-F	Antibody	Lang and Vincent (2009), Gomez et al. (2010)
		Weakness	MuSK, LRP4	L-O-F	Antibody	Vincent and Leite (2005), Cole et al. (2008), ter Beek et al. (2009), Klooster et al. (2012)
	Acquired slow channel syndrome	Weakness	n-AChR, ε	G-O-F	Antibody	Wintzen et al. (1998)
**Peripheral nerve**
Potassium channel	Morvan’s syndrome	Spontaneous muscle movements	VGKC complex, Caspr2	L-O-F	Antibody	Irani et al. (2011)
	Acquired neuromyotonia	Spontaneous muscle movements	VGKC complex, Caspr2	L-O-F	Antibody	Shillito et al. (1995), Newsom-Davis (2004), Irani et al. (2011)
Calcium channel	Lambert–Eaton myasthenic syndrome	Weakness	Ca_v_2.1	L-O-F	Antibody	Lang and Newsom-Davis (1995)

# Loss-of-function (L-O-F) or gain-of-function (G-O-F);

* Selected references (review). ^1^Autosomal dominant; ^2^Autosomal recessive.

(1) All membrane channels of the neuromuscular system, including the AChR channel, have been found to be affected in humans, either by mutation or by autoantibody attack. Some of these channelopathies are rare but others, notably myasthenia gravis, occur more frequently, i.e., 20 per 100,000 (Phillips, 2003).
(2) Given the delicate pattern of ion channel opening and closing it comes as no surprise that inherited changes can lead to aberrant function of the channel, which hampers the essential features of transient inactivation and rapid repolarization. Such changes affect sodium channels and are often of the autosomal dominant type which cause long-lasting depolarization (Jurkat-Rott and Lehmann-Horn, 2004; Jurkat-Rott et al., 2010a,b).
(3) In hereditary channelopathies in muscle the clinical symptoms such as myotonia and paralysis are not present all the time but occur in attacks, whose frequency may vary considerably between patients. Between the attacks the muscle shows a virtually normal performance (Lehmann-Horn and Jurkat-Rott, 1999). Apparently, the phenotype becomes overt only under certain, well-defined changes of environment (potassium and carbohydrate intake) or usage of the muscle.
(4) Although the symptoms of these channelopathies can be largely predicted from the abnormalities observed when these mutations are expressed in single cell systems, uncertainties, and puzzles remain. For instance, myotonia in hyperkalemic periodic paralysis (sodium channel mutation) can have the same pattern as myotonia in Thomsen type chloride channel mutations (Jurkat-Rott et al., 2010a) and for the diagnosis occasionally a genetic analysis is required in order to discriminate between the two diseases. A formidable puzzle has been hypokalemic periodic paralysis where periodic attacks of weakness are associated with low values of blood potassium. Responsible mutations have been found in channels with a completely different function including L-type calcium channels (channels involved in depolarization-contraction coupling in muscle), sodium channels (muscle action potential), and potassium channels (inward rectifier). Nearly all mutations in hypokalemic periodic paralysis are missense mutations at arginine in the voltage sensor S4 transmembrane domain. Each alpha subunit of an ion channel for sodium, potassium, or calcium has six transmembrane segments S1–6 of which S4 contains many positively charged amino acids including arginine. This positive charge is forced to move by the electrical field of the depolarization and the moving charge gives rise to a gating current which causes the channel to open. Mutational substitutions of arginine support an alternative pathway for ion conduction in the form of protons, the gating pore current, and it has been put forward that this could be the cause of the aberrant depolarization during attack of paralysis (Cannon, 2010; Matthews and Hanna, 2010). However, the detailed pathophysiology of this disease has not yet been fully unraveled.
(5) Autoimmune attack usually results in a loss-of-function of the ion channels that are involved (although the terminology of L-O-F is usually reserved for changes caused by genetic mutations). However, the mechanism through which this is brought about can be different. One manner, which might seem obvious, is rare, namely that the autoantibody directly inhibits functioning of the ion channel or blocking it directly. This seems to be the case in acquired neuromyotonia and in some forms of myasthenia gravis (Shillito et al., 1995). More common though, is antigenic modulation where membrane channels or receptors are cross-linked by the antibody, a process that is followed by endocytosis of the channel-antibody complex. In the common type of myasthenia gravis, autoantibody reaction against AChRs also strongly involves activation of complement, leading to local destruction of membrane at the endplate (Gomez et al., 2010; Vincent, 2010). As a result, the muscle membrane regenerates quickly but now with a reduced number of AChRs leading to failing neuromuscular transmission. Similarly, in the Lambert–Eaton myasthenic syndrome, antibodies decrease the density of presynaptic Ca channels (Fukunaga et al., 1983).
(6) As far as we are aware, there is one exception to the general rule that autoantibodies against AChRs lead to L-O-F changes. In the acquired slow channel syndrome antibodies against the epsilon subunit cause the AChR channel to close more slowly which leads to increased inflow of (cytotoxic) calcium ions (Wintzen et al., 1998).
(7) Autoantibodies can be transferred from mother to embryo by placental transfer. Antibodies that cause myasthenia gravis in the pregnant mother may also cause reversible muscle weakness in neonates. Moreover, maternal anti-AChR antibodies may impair skeletal muscle development and cause arthrogryposis multiplex congenita, in which babies are born with multiple joint contractures and muscle weakness. In such cases, the mother is frequently asymptomatic, because maternal antibodies to the gamma subunit of the muscle AChR exclusively bind to the embryonic muscle (Riemersma et al., 1996). In the adult type AChR receptor, the gamma subunit is replaced by an epsilon subunit, thus making the adult muscle resistant to such autoantibodies.
(8) Although ion channels may be affected at the pore-forming subunits, it should be borne in mind that ion channels have channel-associated proteins that are important for their function. Also these are susceptible to mutations and autoimmune attack. For example, mutations in the AChR-associated protein of the synapse (rapsyn) and the muscle specific kinase (MuSK) lead to (recessive) myasthenic syndromes; antibodies against MuSK cause myasthenia gravis (for review, see Gomez et al., 2010).

## CHANNELOPATHIES IN NEUROPSYCHIATRIC DISORDERS

### GENETIC INVOLVEMENT OF ALTERED ION CHANNEL FUNCTIONS IN NEUROPSYCHIATRIC DISORDERS

The pathophysiology of neuropsychiatric disorders like schizophrenia is still poorly understood, although evidence strongly suggests that hypofunction of dopamine receptor and NMDAR and dysfunction of potassium channels play central roles (Stephan et al., 2006; Corti et al., 2011; Vukadinovic and Rosenzweig, 2012). Strong evidence from genetic studies in large (pooled) cohorts has furthermore indicated that certain common and rare genetic variants increase risk for neuropsychiatric disorders including genes encoding components of ion channels. For example, a genome-wide analysis identified risk loci with shared effects on five major neuropsychiatric disorders; this study showed that specific single nucleotide polymorphisms indicated variation in L-type calcium channel gene activity of two subunits, CACNA1C and CACNB2 (Smoller et al., 2013).

Genetic studies have furthermore identified the genetic variants in genes encoding human leukocyte antigen (HLA) regions [major histocompatibility complex (MHC) molecules] as being associated with schizophrenia risk; which is one of the most robust findings in psychiatric genetics to date. This, together with other evidence implicating the HLA DRB1 locus as being associated with risk for schizophrenia and autism, may support the notion for a role for neuroinflammation and possibly autoimmunity in neuropsychiatric disorders (Crespi and Thiselton, 2011). Association with HLA antigens in schizophrenia had been noted before and different authors had already speculated some 20 years ago toward the existence of subgroups of schizophrenics who have immunological abnormalities like antinuclear antibodies in lupus erythematosus, known as neuropsychiatric lupus (Ganguli et al., 1993).

### BLOOD–BRAIN BARRIER AND ACCESS OF AUTOANTIBODIES TO THE CNS

It is a common misconception that the blood–brain barrier (BBB) is completely impermeable to antibodies. In fact, antibodies cross to a limited extent the human BBB (Poduslo et al., 1994). They arrive into the cerebrospinal fluid (CSF) with a rate of 0.018 mg/min and have a turnover of 0.0036/min (comparable to the CSF turnover; four times per day; Poduslo et al., 1994). This very dynamic equilibrium results in immunoglobulin G (IgG) levels in the brain of around 1% of the plasma levels and IgG composes 9.8% of the protein in the CSF (Cutler et al., 1970; Poduslo et al., 1994). There is a large body of evidence that pathogenic autoantibodies (e.g., in autoimmune channelopathies) reach sites in the CNS and have immunopathogenic effects there (Diamond et al., 2009). Such diseases include paraneoplastic neurological syndromes (Honnorat and Antoine, 2007), neuromyelitis optica (Lennon et al., 2004), stiff-person’s syndrome (Solimena et al., 1988), epilepsy with glutamic acid decarboxylase (GAD) antibodies (Solimena et al., 1988; Errichiello et al., 2009), narcolepsy (Cvetkovic-Lopes et al., 2010), dyslexia (Vincent et al., 2002), Morvan’s syndrome, and limbic encephalitis (Irani et al., 2010). Moreover, it has been demonstrated in patients with Alzheimer’s disease and in corresponding AD animal models that intravenous injection of antibodies directed against aggregated amyloid beta efficiently reduce amyloid plaque load in the brain (Panza et al., 2010). The mechanisms by which antibodies pass the BBB under normal conditions are still incompletely understood. The BBB becomes much more permeable following local inflammation reactions, e.g., in multiple sclerosis (0.107 mg/min; Cutler et al., 1970). Moreover, neuroinflammation may lead to local production of antibodies by B-cells and or plasma cells in the brain itself.

However, in spite of this dynamic turnover of antibodies between the periphery and the brain, well-defined autoantibodies of peripheral diseases, such as the Lambert–Eaton myasthenic syndrome and neuromyotonia, do not have central effects despite the fact that calcium Ca_v_2.1 (P/Q-type) channels and potassium channel complex proteins are also present in the brain and thus could be targeted by these antibodies. So in some cases, CNS autoimmune channelopathies occur only when the integrity of the BBB is disrupted and higher flow of antibodies gets access to the brain. From this point of view it might be argued that Morvan’s syndrome and acquired neuromyotonia might harbor the same disease pathophysiology (**Tables 1** and **2**) and the same immunological pathogenesis, with the exception however, that in the case of Morvan’s syndrome there is a spectrum of disease intensities with a breach in the integrity of the BBB. In line with this is the finding that mice immunized with a peptide producing both anti-DNA and anti-NMDAR antibodies do not show brain pathology unless the integrity of the BBB is challenged (Diamond et al., 2009). Because it is unlikely that the BBB is equally permeable to antibodies or antibody-producing cells throughout the brain, the “phenotype” of the antibody effect may well depend on the location where the antibody enters the brain and factors affecting the integrity of the BBB with respect to the transport of antibodies may show regional specificity. This might explain why the same autoantibody might have substantially different pathological effects between different groups of patients. For instance, antibodies against potassium channel complex proteins are involved in limbic encephalitis, Morvan’s syndrome (Irani et al., 2010), or schizophrenia (Zandi et al., 2011); after lipopolysaccharide treatment, anti-NMDAR antibodies attack channels in hippocampal neurons while the same antibodies after treatment with adrenaline, also known to breach BBB integrity, damage channels in other regions of the brain (for a review, see Diamond et al., 2009).

**Table 2:** Ion channel targets of autoantibodies in the brain.

Targeted antigen	Disease(s)	Subunit/associated protein	Reference
Potassium channel/VGKC-complex	Limbic encephalitis, Morvan’s	LG1 or Caspr2	Irani et al. (2010, 2011), Lai et al. (2010)
	syndrome, Schizophrenia	LG1 or Caspr2	Irani et al. (2011), Zandi et al. (2011), Steiner et al. (2013)
NMDAR	Encephalitis, Schizophrenia	NR1	Dalmau et al. (2011), Zandi et al. (2011), Steiner et al. (2013)
AMPAR	Limbic encephalitis		Vincent et al. (2011)
Glutamic acid decarboxylase	Limbic encephalitis, Stiff person syndrome		Vincent et al. (2011), Graus et al. (2010)
Muscarinic AChR	Schizophrenia	M1; M2	Borda et al. (2002, 2004)
Nicotinic AChR	Schizophrenia	α7	Chandley et al. (2009)
GABA_B_R	Limbic encephalitis		Vincent et al. (2011)
Glycine-R	Progressive encephalomyelopathy		Hutchinson et al. (2008)

### GAIN-OF-FUNCTION, LOSS-OF-FUNCTION, NEUROINFLAMMATION, OR NEURODEGENERATION?

In view of what we know about autoimmune channelopathies in the neuromuscular junction to date, it is reasonable to surmise that most CNS autoimmune channelopathies are probably of the loss-of-function type, as happens in myasthenia gravis, Lambert–Eaton mysthenic syndrome, and acquired neuromyotonia where there is loss of activity or number of AChRs, P/Q-type calcium channels, and potassium channels, respectively (Shillito et al., 1995; Lang and Vincent, 2009; Gomez et al., 2010). In addition, it is possible that there is also complement-induced destruction of membrane around the antigen target as happens in myasthenia gravis. This could start degeneration of the neurons (or potentially also glia, astrocytes, or oligodendrocytes) carrying the antigen. Obviously these are important questions to be dealt with eventually because the success of immunosuppressive therapy will depend on whether the antibody-induced defect is reversible.

### AUTOIMMUNE TARGETS IN THE BRAIN AND NEUROPSYCHIATRIC DISORDERS

**Table 2** gives a summary of the ion channel targets for autoantibodies in the brain and different diseases that are related to them. Recently, antibodies to neuronal cell surface antigens have been identified in cases of autoimmune encephalitis that respond to immunotherapy (Zandi et al., 2011). Over two-thirds of patients with NMDAR antibody encephalitis, and some with potassium channel antibody associated limbic encephalitis, have marked psychiatric symptoms, or have their initial presentation at psychiatric services (Creten et al., 2011). The psychiatric symptoms are similar to those seen in schizophrenia including delusions, hallucinations, and catatonic movement disorder. These antibodies seem to be responsible for the pathophysiology because they seem to be absent in healthy individuals, and in the majority of patients with other neurological diseases (Zandi et al., 2011; Steiner et al., 2013). Recently, several case reports described patients with NMDAR or potassium channel antibodies suffering exclusively from neuropsychiatric disorders; and, interestingly, treatment with immunosuppressive agents resulted in long lasting clinical improvement in one of these patients (Zandi et al., 2011; Steiner et al., 2013). Although in one study NMDA antibodies were not detected in a group of 80 patients with schizophrenia (Masdeu et al., 2012), another study did report an increase in the prevalence of diverse NMDA antibodies in patients with initial diagnosis of schizophrenia (Steiner et al., 2013).

Antibodies to muscarinic AChRs (mAChRs) have been identified in a small proportion of a group of schizophrenic patients, and these antibodies were shown to interact with cerebral frontal cortex mAChR, where they react against the second extracellular loop of the M1 and M2 regions (Borda et al., 2002, 2004). Surprisingly, these antibodies had an agonistic, “G-O-F”-like effect on mAChRs, i.e., causing an increase of the cyclic GMP production, accumulation of inositol phosphate, and translocation of protein kinase C (Borda et al., 2002, 2004). Another study was able to identify antibody titers in 23% of a cohort of 21 schizophrenia patients against the alpha7 subunit of the nicotinic AChR (Chandley et al., 2009).

Systemic lupus erythematosus (SLE) is known to be associated with neuropsychiatric manifestations in some cases (Diamond et al., 2009). A subset of antibodies to double stranded DNA (dsDNA) in patients with SLE cross-reacts with subunits of the NMDAR2a and NMDAR2b on neuronal cells and cause neuronal death by “G-O-F” effect on these receptors leading to excitation-toxicity and apoptosis (DeGiorgio et al., 2001; Omdal et al., 2005). Higher serum and CSF anti-NR2 region antibody levels correlate with depressive mood in SLE patients (Lapteva et al., 2006; Yoshio et al., 2006). Similar examples come from obsessive-compulsive disorders where antibodies directed against basal ganglia antigens occur 20% in of the patients compared to 4% in the controls (Nicholson et al., 2012).

Like in neuromuscular diseases, ion channel-associated proteins in the CNS are vulnerable as targets for autoantibodies. The potassium channel is a case in point. Whereas antibodies directed against channel-associated proteins have been shown to immuno-precipitate the solubilized channel in radioimmune assays, in neuromyotonia and also in (limbic) encephalitis, the autoantibody has been shown in diagnostic cell-based assays to be directed not against the potassium channel alpha subunit, but against leucine-rich glioma inactivated 1 (Lgi1) or contactin-associated protein-like 2 (Caspr2) which both are channel-associated proteins (Lai et al., 2010; Dalmau et al., 2011).

Autoimmunity in mental illness is not limited to ion channels. Katzav et al. (2007) found that injection of intracerebroventricular anti-ribosomal P antibodies induced depression-like behavior in mice. A further example of autoimmunity is that antibodies targeting the folate receptor (FR) have been found in early onset low-functioning autism, where these antibodies seem to appear at the age of 4–6 months. FR autoantibodies block the folate binding site of the membrane-attached FR on the choroid epithelial cells, thereby inhibiting the transport of folate into the CSF. In line with a putatively causal relation, changes in FR antibody titer correlated with behavioral incidents, and interestingly, a regimen of oral folic acid supplements was able to reverse or substantially improve the clinical phenotype in some patients, particularly in children diagnosed before 3 years of age (Ramaekers et al., 2005, 2007; Frye et al., 2013).

It is important to keep in mind that mental illness could also have neurodevelopmental origins. Although many neurodevelopmental disorders are thought to have a major genetic component, other components, such as environmental impact and associated gene–environment interactions have also been proposed (van Os et al., 2010). Interestingly, a role for *maternal* autoantibodies has been reported in a few neurodevelopmental phenotypes, e.g., autism, specific language difficulties, and dyslexia (Warren et al., 1990; Vincent et al., 2002, 2003; Dalton et al., 2003; Martin et al., 2008).

Penetration of IgGs into the brain has been proposed to be greater in neonatal rats than in adult rats (Fabian and Hulsebosch, 1989). Consequently it is possible that maternal antibodies might have easier access to receptors in the fetus brain, activate different pathways, or have other effector functions. In addition, the NMDAR, and some other receptors as well, can exist in two forms, embryonic and adult types (Monyer et al., 1994; Fukaya et al., 2005). Thus it is possible that antibodies selective against embryonic forms of the channel subunit are present in an asymptomatic mother and cause a direct effect on the fetal brain during pregnancy. This would be analogous to the situation in arthrogryposis multiplex congenita as explained above for the fetal muscle.

In some cases the auto-antibody-induced damage during brain development may never lead to clinical manifestations or might induce neuropsychiatric manifestations later in life after a second hit, e.g., an environmental trigger. If true this would complicate the diagnosis and investigation of disease pathology of these cases.

All in all, as an increasing number of patients with neuropsychiatric disorders with autoimmune antibodies are being reported, it would be important to start large systematic studies of patients, as well as of offspring and other relatives, in order to examine the occurrence of autoimmune-related pathology such as anti-channel antibodies. In addition, the study of pathogenic antibodies may also enable novel treatment options (using immunosuppressants, plasmapheresis, etc.) for neuropsychiatric disorders.

## Conflict of Interest Statement

The authors declare that the research was conducted in the absence of any commercial or financial relationships that could be construed as a potential conflict of interest.
